# Growth differentiation factor 15: from stress response to clinical utility in chronic liver diseases

**DOI:** 10.1007/s00535-025-02336-7

**Published:** 2025-12-23

**Authors:** Yuta Myojin, Hayato Hikita

**Affiliations:** Department of Gastroenterology and Hepatology, Graduate School of Medicine, The University of Osaka, 2-2 Yamadaoka, Suita, Osaka 565-0871 Japan

**Keywords:** Liver cancer, Biomarker, Hepatic decompensation

## Abstract

The clinical landscape of chronic liver disease has changed with effective antiviral therapies, enabling the eradication of hepatitis C virus and the durable suppression of hepatitis B virus replication. Despite these advances, patients remain at risk for hepatocellular carcinoma (HCC) and other liver-related complications, but the growing burden of metabolic dysfunction-associated steatotic liver disease (MASLD) has created new challenges for clinical practice. These trends emphasize the need for reliable, noninvasive biomarkers that can stratify risk and guide long-term management across diverse etiologies. Growth differentiation factor 15 (GDF15), a stress-inducible cytokine, has attracted increasing interest as a promising biomarker. Its expression is induced by metabolic, oxidative, and inflammatory stress, and circulating levels increase with disease progression. Elevated serum GDF15 is consistently associated with fibrosis severity, HCC risk, hepatic decompensation, and mortality. Importantly, GDF15 is not merely a surrogate of fibrosis; rather, it integrates hepatocellular and stromal stress pathways and captures residual risk beyond fibrosis stage, liver function scores, and conventional biomarkers. In addition to its prognostic association, GDF15 has diverse biological effects. It may act as a protective response by limiting inflammation and cellular injury; yet, in other contexts, it contributes to fibrogenesis, tumor progression, immunosuppression, and cachexia. These dual roles highlight both the potential and the complexity of targeting GDF15 in therapeutic strategies. Collectively, the results of the current study indicate that GDF15 represents a promising biomarker in chronic hepatic diseases and is clinically independent of hepatic fibrosis. Further work is needed to clarify the underlying mechanisms, validate the prognostic utility, and determine whether GDF15 can be developed as a therapeutic target within precision medicine approaches.

**Supplementary Information:**

The online version contains supplementary material available at 10.1007/s00535-025-02336-7.

## Introduction: clinical need for biomarkers in chronic liver disease

**Fig. 1:**
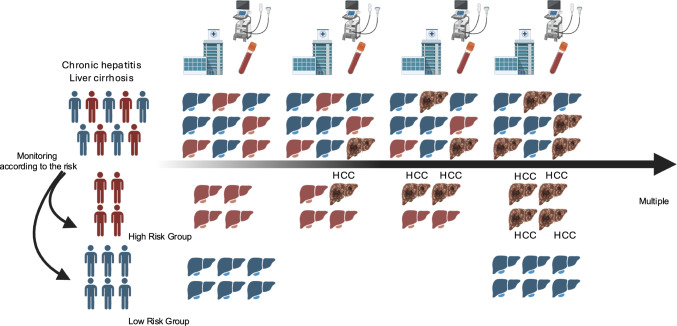
Biomarker-based monitoring in chronic liver disease. Stratifying patients by risk enables more intensive surveillance—more frequent imaging and blood tests—for those at high risk of HCC or adverse outcomes, while allowing reduced testing intervals for low-risk patients

With the advancement of antiviral therapies, the clinical landscape of chronic liver disease has dramatically changed [1, 2]. Hepatitis C virus (HCV) can now be eradicated in most patients with direct-acting antivirals (DAAs), and hepatitis B virus (HBV) replication can be effectively suppressed with nucleotide analogs (NAs) [3, 4]. Nevertheless, even when receiving these treatments, patients still have a risk for hepatocellular carcinoma (HCC) [5–7], and their long-term outcomes are heterogeneous and depend on multiple factors [7, 8]. In addition, the growing burden of MASLD presents new challenges in clinical practice, particularly regarding how frequently these patients should be monitored for disease progression and HCC development [1, 9–12]. Especially in MASLD, the most important risk factor is liver fibrosis [13, 14], and several noninvasive liver disease assessments (NILDAs) for fibrosis are now available [15, 16]. However, although the frequency is lower than that in advanced fibrosis, patients with nonadvanced fibrosis can still develop HCC [17]. Considering the large number of patients with nonadvanced fibrosis, reliable, noninvasive biomarkers independent of fibrosis that can stratify prognosis, identify high-risk subgroups, and optimize routine imaging surveillance are urgently needed (Fig. 1). Growth differentiation factor 15 (GDF15) was recently reported to be a promising candidate with potential prognostic value in this context [17–19]. GDF15 is not simply a “fibrosis marker” but also a panhepatic stress indicator—an integrated signal of hepatocyte injury, immune-stromal activation, and metabolic stress that offers prognostic information beyond fibrosis metrics and standard scores [17, 18, 20]. In this review, we summarize mechanistic insights and evaluate the prognostic potential of GDF15 in chronic liver disease.

## Biology of GDF15

**Fig. 2:**
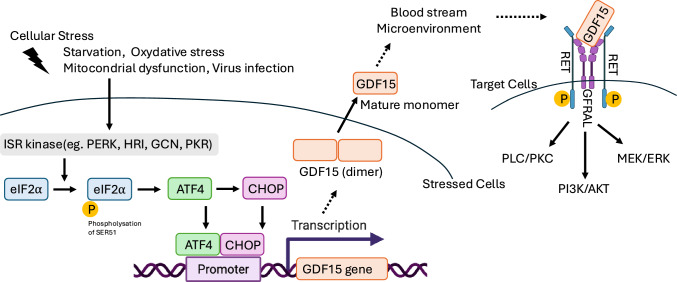
GDF15 as a stress cytokine. Diverse stressors (starvation, oxidative stress, mitochondrial dysfunction, viral infection) activate ISR kinases (PERK, GCN2, HRI, PKR), which phosphorylate eIF2α (Ser51) and promote selective translation of ATF4 with induction of CHOP. The ATF4-CHOP axis drives GDF15 transcription. Newly synthesized GDF15 is processed and secreted into the microenvironment and bloodstream, where it acts on target cells via the GFRAL–RET receptor complex, engaging downstream PI3K/AKT, MEK/ERK, and PLC/PKC pathways

GDF15 is a stress-induced cytokine that was originally identified as an autocrine inhibitor of macrophage activation in the placenta and named macrophage inhibitory cytokine-1 (MIC-1) [21, 22]. A distant member of the transforming growth factor-beta (TGF-β) superfamily, GDF15 has emerged as a key cytokine involved in cellular adaptation to a wide range of stress conditions [23]. Upon exposure to stressors such as nutrient deprivation [24], oxidative stress [25], mitochondrial dysfunction [26], or viral infection [27], the integrated stress response (ISR) is activated through the phosphorylation of eukaryotic initiation factor 2 alpha (eIF2α) by ISR kinases, including PERK, GCN2, PKR, and HRI [28]. Phosphorylated eIF2α leads to the selective translation of ATF4, which subsequently induces CHOP expression. ATF4 and CHOP form a heterodimer that binds to the promoter region of the GDF15 gene [23], thereby enhancing its transcription. The GDF15 protein is secreted into the extracellular space and enters the bloodstream, where it reaches distant target cells (Fig. 2). As a secreted cytokine, GDF15 functions in autocrine, paracrine, and endocrine signaling [22, 29, 30]. One of its well-known features is its systemic signaling via the glial cell-derived neurotrophic factor receptor alpha-like (GFRAL) receptor, which is expressed in the area postrema and nucleus tractus solitarius of the hindbrain [31–34]. This endocrine effect allows GDF15 to influence appetite regulation, energy expenditure, and systemic metabolism [35–37]. GDF15 binds to the GFRAL–RET receptor complex, thereby activating downstream signaling pathways, including various kinase cascades involved in metabolic regulation, inflammation [38], and cancer cell proliferation [30, 39]. FGF21 and GDF15 are ISR-inducible mitokines with different targets: FGF21 (FGFR1c/beta-Klotho) drives adaptive metabolic responses, while GDF15 (GFRAL–RET) relays mitochondrial stress to the brainstem to suppress appetite [23].

## Conditions associated with elevated peripheral GDF15

**Fig. 3:**
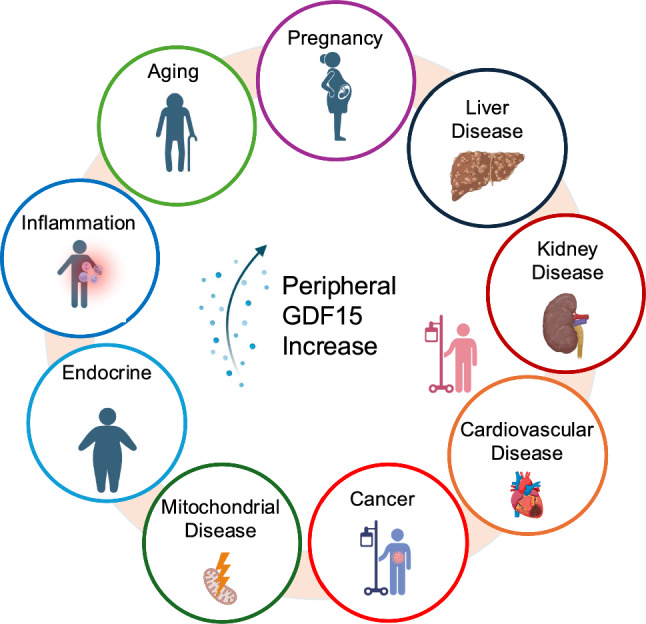
Circulating GDF15 increases in various conditions. Peripheral GDF15 rises across a range of endogenous stress-related states, including pregnancy, liver disease, kidney disease, cardiovascular disease, cancer, mitochondrial disorders, diabetes mellitus, inflammatory conditions, and aging. A summary is provided in Table 1, with detailed disease-specific values and study characteristics in Supplementary Table 1

As cellular stress induces GDF15 expression, peripheral blood GDF15 levels are elevated across diverse conditions (Fig. 3, Table 1, Supplementary Table 1). We describe the changes in peripheral GDF15 levels under multiple conditions in detail in Supplementary Table 1.

**Table 1:** Circulating GDF15 increases under various conditions

Condition	Change	GDF15 trend and clinical implications	References
Pregnancy	Peripheral GDF15 increases in pregnancy	The placenta secretes GDF15, which is involved in immune tolerance. GDF15 levels increase as pregnancy progresses	[43–46]
Aging	Peripheral GDF15 levels increase with aging	Even in healthy individuals, GDF15 levels increase with age, reflecting the accumulation of biological stress responses	[40, 41]
Inflammation	Peripheral GDF15 increases in infection and systemic autoimmune disease	Peripheral GDF15 levels are elevated in infectious and autoimmune diseases, serving as an indicator of systemic organ injury and prognosis	[87–93, 95–101]
Endocrine	GDF15 levels in patients with diabetes mellitus are higher than those in heathy controls	GDF15 levels are related to cardiovascular risk and prognosis	[53–61]
Mitochondrial disease	Peripheral GDF15 levels increase in mitochondrial disease	GDF15 is markedly elevated in mitochondrial disease and can be used as a diagnosis biomarker	[47–49, 51, 52]
Liver disease	Peripheral GDF15 levels are higher in patients with chronic liver disease than in healthy controls	GDF15 levels increase as liver disease progress. GDF15 levels are related to cancer occurrence and prognosis	[17–20, 83–86, 124]
Kidney disease	Peripheral GDF15 levels are negatively correlated with eGFR	GDF15 levels increase as kidney disease progresses as a stress response. GDF15 levels are related to the mortality	[41, 82]
Cardiovascular disease	Peripheral GDF15 levels increase in cardiovascular events	Circulating GDF15 levels are significantly elevated in acute coronary syndrome and in patients with heart failure. Importantly, GDF15 has been linked to adverse left ventricular remodeling and poorer prognosis	[54, 62–66, 68–81]
Cancer	Peripheral GDF15 levels increase in multiple cancers	Circulating GDF15 levels are markedly elevated across multiple malignancies. Elevated GDF15 has been linked to adverse prognosis and to the development of cancer-associated cachexia	[102–122, 146, 149–151]
Other		GDF15 is related to the disease severity and prognosis	[152–158]

Among healthy individuals, GDF15 increases with age—from ~ 0.54 to 0.63 ng/mL in those < 30 years to ~ 1.85 to 2.15 ng/mL in those ≥ 80 years [40, 41].

During pregnancy, the levels of circulating GDF15 increase dramatically, because the fetoplacental unit is the dominant source; the levels increase from early gestation and peak in the late third trimester, with a subsequent and rapid decrease postpartum [42–46]. In patients with gestational diabetes, the medians are greater and are correlated with glycemic indices [45]. Compared with that in controls, serum GDF15 levels in pediatric patients with mitochondrial diseases are consistently and markedly elevated [47–52]. In patients with endocrine disorders, especially diabetes mellitus, circulating GDF15 levels increase and are related to cardiometabolic risk [53, 54], poor peripheral nerve condition [55–58], and increased risk of peripheral artery disease [59, 60]. GDF15 is elevated in other endocrine diseases, such as hyperthyroidism, and decreases after antithyroid therapy [61]. Cardiovascular cohorts show increases in the expression of cardiac injury markers in acute coronary syndrome [62, 63], which are related to prognosis and severity [64–68]. Following ablation of atrial fibrillation, GDF15 levels support the prediction of recurrence [69]. In cardiovascular surgery, GDF15 can predict peri-surgery risks [70, 71] and is related to the postsurgical outcome [72–75]. In end-stage heart failure, GDF15 levels are elevated and can predict prognosis [76–78]. GDF15 levels indicate the severity of pediatric unrepaired shunt chronic heart disease [79] and are useful for the diagnosis of pulmonary hypertension [80]. In patients with newly diagnosed essential hypertension, circulating GDF15 levels are significantly high in nondippers, which is linked to increased cardiovascular risk [81]. In kidney disease [41, 82], chronic liver diseases [17–20, 83–86], infectious diseases [87–90], and immune-mediated inflammatory diseases [91–101], the levels of circulating GDF15 are consistently elevated compared with those in controls. Compared with that in controls, circulating GDF15 is consistently elevated in patients with solid and hematologic malignancies, such as in breast cancer [102, 103], colorectal cancer [104–106], hepatocellular carcinoma [107, 108], lung cancer [109], melanoma [110–112], pancreatic cancer [113–119], and prostate cancer [120, 121]. Collectively, GDF15 is a broadly useful—although not tumor-specific—serologic marker that complements conventional assays for detection, risk stratification, therapy monitoring, and prognostication in cancer [122].

Since cancer stage, population, and assay methodology differ among studies, these values should not be used for direct cross-disease comparisons. Nevertheless, notably in patients with chronic liver disease, elevated GDF15 is not necessarily liver event-specific and may reflect extrahepatic comorbidity, age, renal function, or treatment effects.

## Blood GDF15 levels in liver diseases

**Fig. 4:**
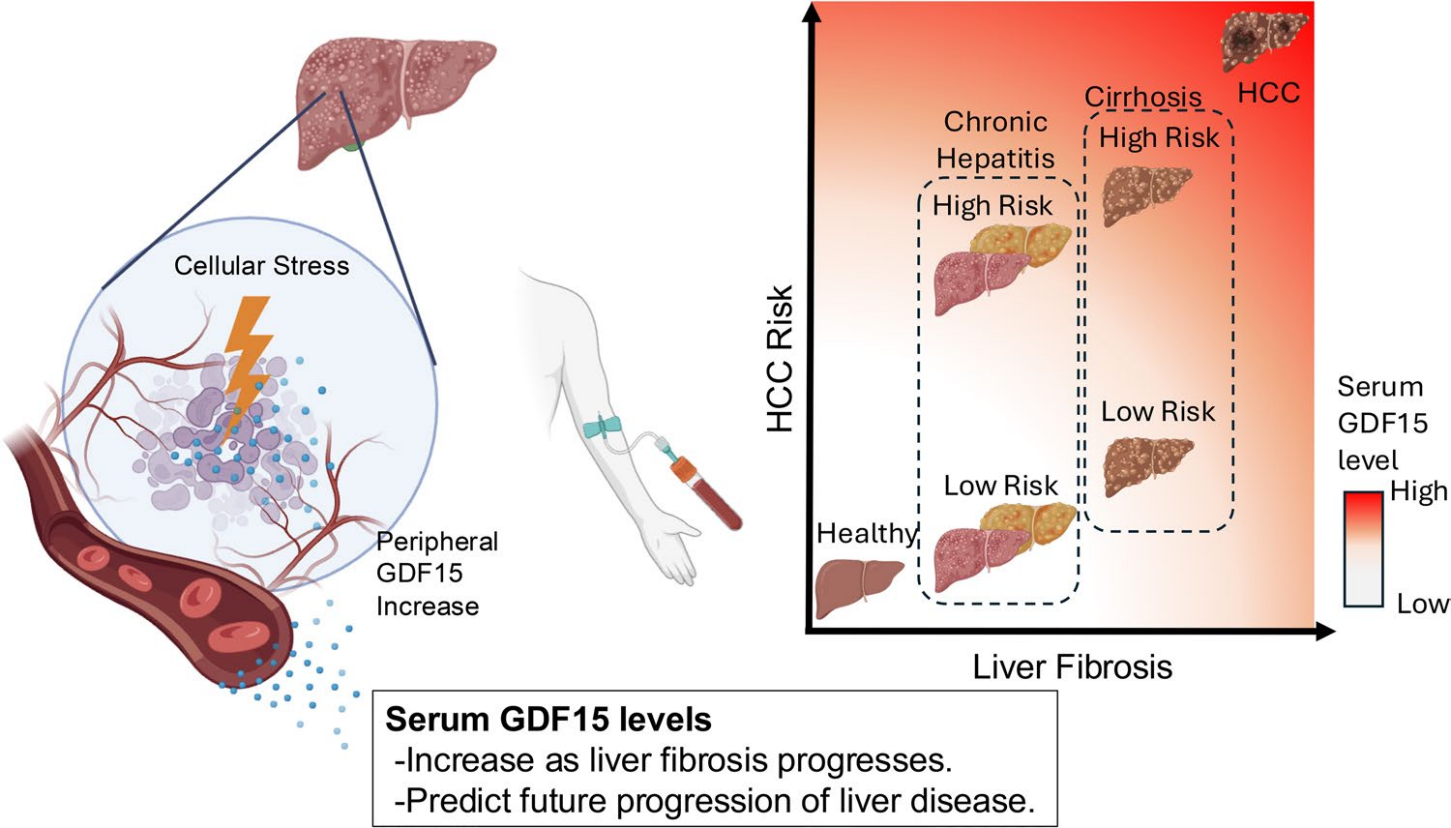
GDF15 as a stress marker in chronic liver disease. In chronic liver disease, hepatic cellular stress induces GDF15 production and secretion, leading to elevated levels in the peripheral circulation. GDF15 level reflects both HCC risk and liver fibrosis

In chronic liver diseases, such as chronic hepatitis B [19], chronic hepatitis C [18], and MASLD [17, 86, 123], auto immune hepatitis, and primary biliary cholangitis [124], the levels of circulating GDF15 are consistently elevated. Compared with that in controls, the expression of GDF15 in patients with cirrhosis and hepatocellular carcinoma is greater and increases with increasing fibrosis burden [30]. Among patients with HCV-related cirrhosis after viral eradication, baseline levels remain high, which is consistent with advanced disease [20]. In MASLD, median levels cluster near the 1 ng/mL range (e.g., ~ 1.23 ng/mL) and are higher in biopsy-defined steatohepatitis than in MASLD without MASH [85]. In people with HIV, GDF15 expression is also higher in those with MAFLD than in those without MAFLD [86]. Taken together, these data indicate that blood GDF15 levels increase across etiologies—from viral hepatitis and MASLD to cirrhosis and HCC—and track the transition from steatosis to steatohepatitis, fibrotic remodeling, and malignant transformation by cellular stress (Fig. 4).

## GDF15 in liver pathophysiology

The liver is uniquely susceptible to metabolic and inflammatory stressors because of its central role in nutrient metabolism, detoxification, and immune surveillance [125]. The role of GDF15 in hepatic pathophysiology is being increasingly examined through its context-dependent actions in various liver cell populations. In hepatocytes, GDF15 is upregulated by mitochondrial stress, lipid overload, and proinflammatory cytokines [126, 127]. In MASLD, lipid accumulation leads to lipotoxicity, oxidative stress, and ER stress, all of which are potent inducers of GDF15 expression [128]. Macrophages also contribute to GDF15 production under fibrogenic and inflammatory stimuli and to lipid accumulation [126, 129]. In addition to promoting autophagy in cancer cells, hepatic stellate cells also secrete GDF15 and promote liver cancer progression via paracrine effects [30, 130]. Immunohistochemistry of human hepatocellular carcinoma showed GDF15 predominantly in hepatic stellate cells and macrophages—rather than tumor cells—with higher staining in tumor than adjacent non-tumor liver [30]. Based on this pattern, circulating GDF15 is supposed to decrease after effective tumor-directed therapy.

GDF15 may function as a protective response for hepatocytes, modulating apoptotic pathways, dampening inflammation, and curbing excessive lipid accumulation [131]. Conversely, GDF15 has been associated with profibrogenic signaling and protumor signaling. Some studies have suggested that GDF15 can activate hepatic stellate cells through the SMAD signaling system, enhancing extracellular matrix production [34, 132]. These paradoxical behaviors highlight the complexity of GDF15 biology, which may vary depending on disease stage, etiology, and cellular context.

## Blood GDF15 as a biomarker in chronic liver diseases

As outlined above, GDF15 has emerged as a promising noninvasive biomarker for predicting HCC and other liver-related events in patients with chronic liver disease. HCC remains among the most lethal complications and most often arises in the context of cirrhosis or persistent inflammation [20]. Reliable biomarkers for early HCC detection and risk stratification are needed. GDF15 is dynamically upregulated along the fibrosis–hepatocarcinogenesis axis, and some studies have shown that elevated serum GDF15 levels precede HCC development in patients with chronic hepatitis or cirrhosis [17–19]. Notably, among patients who achieved a sustained virologic response (SVR) after HCV therapy, higher baseline GDF15 expression was associated with an increased risk of de novo HCC (HR 2.54, *p* = 0.0287 after adjustment for AFP, FIB-4 index, and ALBI score) [18]. In patients with HBV, those with higher GDF15 levels had a greater risk for HCC occurrence than those with lower GDF15 levels did (HR 1.62, *p* = 0.0107 after the adjustment for sex, GGT, HBsAg, AFP, and the Fib4 index) [19]. In addition, in the context of HCC surveillance, combining GDF15 with established markers such as the Fib4 index and AFP can increase diagnostic accuracy [5, 7, 18, 133].

Across etiologies, higher GDF15 levels are consistently associated with incident HCC and liver-related events independent of fibrosis surrogates (e.g., FIB-4), liver function (e.g., ALBI, Child–Pugh), and AFP [17–20]. In MASLD, GDF15 can be used to identify high-risk patients regardless of fibrosis stage [17, 18]; in patients with HCV-related cirrhosis, it can predict decompensation and mortality following conditioning according to the Child–Pugh [20] score. These results support GDF15 as an orthogonal “stress indicator” that summarizes cumulative hepatic injury and systemic stress rather than merely reflecting fibrosis burden.

In addition to its association with HCC, GDF15 is strongly associated with liver-related clinical outcomes, including hepatic decompensation events such as ascites, encephalopathy, and variceal bleeding. Observational studies have reported that elevated GDF15 levels are linked to an increased risk of liver-related hospitalization (HR 3.85, *p* = 0.0259 after conditioning according to the Child–Pugh score) and mortality, with no deaths observed in the GDF15-low group among patients with HCV-related cirrhosis [20]. These associations held in the MASLD cohort, predicting liver-related hospitalization and mortality [17], suggesting that GDF15 levels reflect residual risk independent of routine clinical parameters such as the Fib4 index, Child–Pugh score, and AFP level. The prognostic utility of GDF15 in mortality might be due to its ability to reflect subclinical hepatic stress and systemic dysfunction, as we reviewed in Table 1, such as inflammation [38], sarcopenia [134, 135], and cardiovascular comorbidities [136, 137]. Moreover, its predictive value extends beyond liver-specific outcomes, as GDF15 has been shown to be a robust marker of all-cause mortality in patients with chronic liver disease [17, 30]. These findings highlight GDF15 as a novel biomarker with broad applicability in chronic liver disease management.

## GDF15 as a therapeutic target in liver disease

Given its role in liver disease, GDF15 has attracted interest not only as a biomarker but also as a therapeutic target. However, its therapeutic application might be challenging because of its pleiotropic effects and context-dependent functions.

**Fig. 5:**
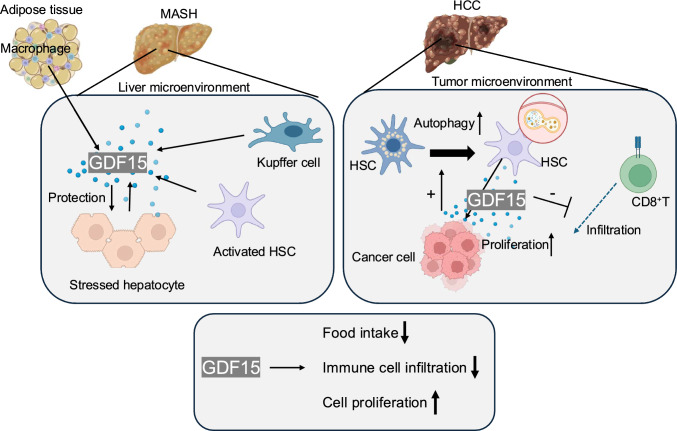
GDF15 in liver microenvironment. GDF15 may represent a novel therapeutic target in MASH and HCC. In MASH, GDF15 is secreted by adipose tissue macrophages, stressed hepatocytes, Kupffer cells, and hepatic stellate cells, and may reduce food intake and immune cell infiltration while helping protect hepatocytes from ROS-induced injury. In contrast, in HCC, GDF15 appears to play key roles in cancer cachexia and tumor progression and may suppress intratumoral CD8⁺ T cell infiltration

Targeting the GDF15–GFRAL pathway to suppress appetite represents a promising strategy for weight reduction in obesity and metabolic disease [128, 138]. Preclinical work with pharmacologic GDF15, including recombinant proteins and receptor agonists, has consistently shown the suppression of food intake and loss of body weight in animal models [31, 37, 126, 139]. Most weight reduction reflects decreases in fat mass with relative preservation of lean tissue. Mechanistically, these effects are mediated by hindbrain GFRAL signaling, which not only reduces appetite but also increases energy expenditure, in part through beta-adrenergic pathways that promote fatty acid oxidation in skeletal muscle [31, 140]. In humans, a long-acting recombinant GDF15 analog (MBL949) has been studied in randomized, placebo-controlled trials. A phase 1 study supported acceptable safety and revealed dose-related signals of weight loss in individuals with overweight or obesity. However, a phase 2 study using biweekly dosing over 14 weeks yielded modest average weight loss, while overall tolerability remained favorable; gastrointestinal symptoms were the most frequent adverse events [141]. Such strategies may benefit patients with MASLD, where obesity and insulin resistance are central drivers of the disease (Fig. 5 left).

On the other hand, elevated GDF15 has been linked to cancer progression and cachexia, raising concerns about potential adverse effects. In patients with HCC or cirrhosis, excessive GDF15 may drive muscle wasting and immune suppression, contributing to tumor progression.

Experimental evidence suggests that GDF15 neutralization enhances T cell adhesion and promotes intratumoral trafficking. When combined with an immune checkpoint inhibitor, anti-PD-1, GDF15 blockade exhibited synergistic antitumor activity in preclinical models through the recruitment of T cells to tumors [142]. GDF15 has also been reported to increase the suppressive activity of regulatory T cells via CD48 in HCC [143]. GDF15 is a mediator of cancer anorexia–cachexia via the brainstem receptor GFRAL–RET. Neutralization of this pathway has shown clinical benefit. In a 12-week, randomized, double-blind phase 2 trial enrolling 187 patients with cancer cachexia and elevated GDF15 concentration (≥ 1.5 ng/mL), compared with placebo, the anti-GDF15 antibody ponsegromab resulted in significantly greater weight gain, with improvements in appetite, cachexia symptoms, and physical activity [144] (Fig. 5 right).

Consistently, increased levels of circulating GDF15 have been linked to poor prognosis in lung cancer [145] and renal cancer patients treated with immunotherapy [146, 147], supporting the hypothesis that targeting GDF15 may improve immunotherapy efficacy. Recently, in a phase 1–2 clinical trial (GDFATHER-1/2a trial), Melero et al. reported the combination therapy of anti-GDF15 and nivolumab for solid cancer, including HCC, and reported a deep response [148].

## Future perspectives

GDF15 has already emerged as a robust biomarker across diverse conditions, where it reflects biological stress and is associated with prognosis. In gastroenterology, however, further validation is warranted. Large, prospective, multiethnic cohorts are needed to confirm the prognostic performance of GDF15 across different etiologies and disease stages. Standardization of assay platforms and agreement on clinically meaningful thresholds will be essential for its integration into clinical practice. Moreover, combining GDF15 with other biomarkers, imaging modalities, and established clinical scores may enhance its value for risk stratification and surveillance in patients with chronic liver disease and related conditions.

Beyond its clinical utility as a biomarker, mechanistic investigations are needed to clarify how GDF15 affects hepatoprotective and tumor-promoting pathways. Such studies are crucial to understanding its context-dependent role in gastrointestinal diseases. In parallel, early-phase trials exploring the pharmacologic modulation of GDF15 highlight its potential as a therapeutic target. Nevertheless, careful evaluation of safety, efficacy, and patient selection is critical before clinical translation. Together, these efforts will help determine whether GDF15 can be developed not only as a prognostic marker but also as a therapeutic strategy in gastroenterology.

## Conclusion

GDF15 is emerging as a novel and key mediator in chronic liver disease. Its expression reflects hepatic stress, and circulating levels provide important clues to disease progression, cancer risk, and patient outcomes. As a biomarker, GDF15 may help refine risk stratification, guide surveillance strategies, and inform therapeutic decision-making.

Moreover, the therapeutic potential of targeting GDF15 remains complex. Its effects vary depending on the disease, underscoring the need for cautious and carefully designed approaches. Future studies should aim to clarify the mechanisms underlying its protective and pathogenic effects, evaluate how to incorporate GDF15 into clinical practice, and assess its relevance across different liver disease phenotypes.

GDF15 has the potential to be used in biomarker-based precision medicine, where molecular insights inform patient-tailored approaches. Further work is needed to determine whether it can serve as a novel target in disease management.

## Supplementary Information

Below is the link to the electronic supplementary material.

Supplementary file1 (PDF 97 KB)

## Abbreviations

ALT: Alanine aminotransferase
AECOPD: Acute exacerbation of chronic obstructive pulmonary disease
ACS: Acute coronary syndrome
AFP: Alpha-fetoprotein
AF: Atrial fibrillation
ALBI: Albumin–bilirubin score
AMI: Acute myocardial infarction
APS: Antiphospholipid syndrome
ASCVD: Atherosclerotic cardiovascular disease
ATF4: Activating transcription factor 4
AUC: Area under the receiver operating characteristic curve
BAFF: B-cell-activating factor
BPH: Benign prostatic hyperplasia
CABG: Coronary artery bypass grafting
CAD: Coronary artery disease
CA15-3: Cancer antigen 15-3
CA19-9: Carbohydrate antigen 19-9
CA125: Cancer antigen 125
CH-B: Chronic hepatitis B
CH-C: Chronic hepatitis C
CHOP: C/EBP homologous protein (DDIT3)
COPD: Chronic obstructive pulmonary disease
CP: Child–Pugh
CRP: C-reactive protein
CRC: Colorectal cancer
CSF: Cerebrospinal fluid
DAA: Direct-acting antiviral
DPN: Diabetic peripheral neuropathy
eGFR: Estimated glomerular filtration rate
eIF2α: Eukaryotic initiation factor 2 alpha
ELISA: Enzyme-linked immunosorbent assay
ER: Endoplasmic reticulum
ESR: Erythrocyte sedimentation rate
ESSDAI: EULAR Sjögren’s syndrome disease activity index
FGF21: Fibroblast growth factor 21
FIB-4: Fibrosis-4 index
FPG: Fasting plasma glucose
GCN2: General control nonderepressible-2 (EIF2AK4)
GDF15: Growth differentiation factor 15
GDM: Gestational diabetes mellitus
GFRAL: GDNF family receptor alpha-like
GI: Gastrointestinal
GGT: Gamma-glutamyl transpeptidase
HAIC: Hepatic arterial infusion chemotherapy
HBV: Hepatitis B virus
HBsAg: Hepatitis B surface antigen
HCC: Hepatocellular carcinoma
HCV: Hepatitis C virus
HD: Hemodialysis
HFpEF: Heart failure with preserved ejection fraction
HIV: Human immunodeficiency virus
HRI: Heme-regulated eIF2α kinase (EIF2AK1)
HR: Hazard ratio
HSC: Hepatic stellate cell
ICI: Immune checkpoint inhibitor
ICU: Intensive care unit
ILD: Interstitial lung disease
IMT: Intima-media thickness
ISR: Integrated stress response
IVIG: Intravenous immunoglobulin
KD: Kawasaki disease
LC: Liver cirrhosis
LEAD: Lower extremity atherosclerotic disease
LNM: Lymph-node metastasis
LTIA: Latex turbidimetric immunoassay
LV: Left ventricular
MAFLD: Metabolic dysfunction-associated fatty liver disease
MASH: Metabolic dysfunction-associated steatohepatitis
MASLD: Metabolic dysfunction-associated steatotic liver disease
MIC-1: Macrophage inhibitory cytokine-1
MRI: Magnetic resonance imaging
mtDNA: Mitochondrial DNA
NA/NUCs: Nucleos(t)ide analogs
NCV: Nerve conduction velocity
NIHSS: National institutes of health stroke scale
NSCLC: Non-small cell lung cancer
NTS: Nucleus tractus solitarius
OPN: Osteopontin
OS: Overall survival
OSCC: Oral squamous cell carcinoma
PAH: Pulmonary arterial hypertension
PC: Prostate cancer
PD: Parkinson’s disease
PDAC: Pancreatic ductal adenocarcinoma
PE: Pre-eclampsia
PERK: Protein kinase R-like ER kinase (EIF2AK3)
PFS: Progression-free survival
PKR: Protein kinase R (EIF2AK2)
PSA: Prostate-specific antigen
pSS: Primary Sjögren’s syndrome
RCC: Renal cell carcinoma
RDS: Respiratory distress syndrome
RET: Rearranged during transfection (proto-oncogene)
ROC: Receiver operating characteristic
SAA: Severe aplastic anemia
SLE: Systemic lupus erythematosus
SpA: Spondyloarthritis
SVR: Sustained virologic response
SYNTAX: SYNergy between PCI with TAXus and cardiac surgery (angiographic score)
T2DM: Type 2 diabetes mellitus
TACE: Transarterial chemoembolization
TDβT: Transfusion-dependent β-thalassemia
TFF3: Trefoil factor 3
TGF-β: Transforming growth factor-β
UM: Uveal melanoma
UPDRS-III: Unified Parkinson’s disease rating scale part III
