# Supplementary material for: Growth differentiation factor 15: from stress response to clinical utility in chronic liver diseases
Source: J Gastroenterol. 2025 Dec 23;61(2):117–30. doi: 10.1007/s00535-025-02336-7 (PMC12924844; doi:10.1007/s00535-025-02336-7)
Supplement: Supplementary file 1 — Supplementary file1 (PDF 97 KB) [file 535_2025_2336_MOESM1_ESM.pdf]

Supplementary Table1. Peripheral GDF15 level and multiple diseases

	Organ	Disease	Aim to measure GDF15 level	n	Control condition	Control GDF	Disease condition 1	GDF15((ng/m	Disease condition 2	GDF15(	Disease condition 3	GDF15((ng	summary	Reffrence #	ref
2	Pregnancy	Gestational diabetes mellitus	To evaluate whether circulating GDF-15 differs between GDM and NGT and relates to glucose metabolism indices in late pregnancy	n=660	Control	13.941	Gestational diabetes mellitus	18.462					Maternal GDF-15 is higher in GDM and correlates with glycemic indices	[45]	Serum growth differentiation factor 15 is associated with glucose metabolism in the third trimester in Chinese pregnant women
2	Pregnancy	Pre-eclampsia	To localize MIC-1/GDF-15 in gestational tissues across pregnancy and determine how maternal serum levels vary with gestation, labor, and pre-eclampsia.	n=194	Normal pregnancy 6–24 weeks	11.4	3–35 weeks	38					Maternal serum GDF-15 rises with advancing gestation, shows no significant change with labor or pre-eclampsia, while tissue staining confirms broad placental/decidual expression throughout pregnancy.	[46]	Macrophage Inhibitory Cytokine-1 in Gestational Tissues and Maternal Serum in Normal and Pre-eclamptic Pregnancy.
2	Pregnancy	Pre-eclampsia	To determine if maternal serum GDF-15 changes in 2nd/3rd trimester and predicts/associates with pre-eclampsia.	n=409	Control	35.11	Pre-eclampsia	41.13					GDF-15 rose across gestation and was significantly higher in preterm PE at 30–34 weeks versus controls, suggesting placental stress signal utility.	[43]	Second- and third-trimester serum levels of growth-differentiation factor-15 in pre-eclampsia.
2	Pregnancy	Pre-eclampsia	To test whether circulating GDF15 is altered at PE presentation (and by subtype) and whether first-trimester levels predict late-onset PE.	n=198	Control	59.776	Pre-eclampsia	20.56					GDF15 rises with normal gestation but is significantly reduced at PE presentation	[44]	Serum levels of GDF15 are reduced in preeclampsia and the reduction is more profound in late-onset than early-onset cases
3	Mitochondrial Disease	Mitochondrial Disease	To evaluate whether serum GDF15 (alongside FGF21) discriminates mitochondrial myopathy from other conditions and how it varies by genetic subgroup.	n=122	Control	0.328	Mitochondrial disease	2.146					GDF15 is markedly elevated in mitochondrial myopathy—especially with translation defects and mtDNA deletions—and correlates with FGF21, but it is less specific than FGF21	[50]	FGF21 is a biomarker for mitochondrial translation and mtDNA maintenance disorders
3	Mitochondrial Disease	Mitochondrial diseases	To evaluate whether circulating GDF-15 can aid screening/diagnosis of pediatric PMDs and assess links with phenotype/genotype and disease severity,	n=131	Control	0.221	Primary mitochondrial disorders	0.919	non-mitochondrial neuromuscular diseases	0.294			Best diagnostic performance among tested biomarkers for PMDs	[47]	Circulating FGF21 and GDF15 as Biomarkers for Screening, Diagnosis, and Severity Assessment of Primary Mitochondrial Disorders in Children
3	Mitochondrial Disease	Mitochondrial diseases	To directly compare GDF-15 against FGF-21 for diagnosing mitochondrial disease in the same adult cohort.	n=140	Control	1.184	Mitochondrial disease	3.956					A comparison of current serum biomarkers as diagnostic indicators of mitochondrial diseases.	[49]	A comparison of current serum biomarkers as diagnostic indicators of mitochondrial diseases.
3	Mitochondrial Disease	Mitochondrial diseases	To compare GDF-15 with FGF21 for diagnosing pediatric mitochondrial disease versus healthy and non-MD disease controls.	n=190	Control	0.439	Mitochondrial disease	1.336					Although GDF-15 is elevated in pediatric MD, it is also high in several non-MD conditions; FGF21 shows better overall diagnostic performance when disease controls are included	[51]	FGF21 outperforms GDF15 as a diagnostic biomarker of mitochondrial disease in children.
3	Mitochondrial Disease	Mitochondrial diseases	To evaluate GDF-15 (vs FGF-21) as a diagnostic serum biomarker for pediatric mitochondrial disease and its induction by mitochondrial dysfunction.	n=111	Control	0.35	Mitochondrial disease	4.046					GDF-15 was 11-fold higher in mitochondrial disease and showed good diagnostic performance (AUC ≈ 0.82; cut-off 0.55 ng/mL: sensitivity 67.8%, specificity 92.3%),	[48]	GDF-15 Is Elevated in Children with Mitochondrial Diseases and Is Induced by Mitochondrial Dysfunction.
3	Mitochondrial Disease	Mitochondrial diseases	To evaluate whether GDF-15 (and FGF-21) can sensitively diagnose mitochondrial disorders versus disease and healthy controls.	n=102	Control	0.943	Mitochondrial disease	4.085					Serum GDF-15 is markedly elevated in mitochondrial disorders and discriminates patients from controls.	[52]	Serum fibroblast growth factor 21 and growth differentiation factor 15: Two sensitive biomarkers in the diagnosis of mitochondrial disorders.
4	Endocrine	Childhood obesity	To test whether circulating GDF15 differs between obese and normal-weight children and relates to metabolic and cardiac parameters.	n=59	Control	0.307	Obese	0.314					Serum GDF15 did not differ between groups, but across all children it correlated positively with CRP, ALT, and aortic dimension, suggesting links to inflammation and early cardiac remodeling risk.	[53]	The Relationship between Metabolic Parameters, Cardiac Parameters and MIC-1/GDF15 in Obese Children.
4	Endocrine	Diabetes mellitus	To determine whether serum GDF15 is elevated in new-onset T2D and independently associates with 10-year ASCVD risk	n=107	Control	0.21	T2DM	0.643					GDF15 was significantly higher in T2D and independently correlated with higher Framingham and Pooled Cohort ASCVD risk scores after adjustment for age, LDL-C, and BMI, supporting its utility as a cardiometabolic risk biomarker in new T2D.	[54]	Association between Growth Differentiation Factor 15 (GDF15) and Cardiovascular Risk in Patients with Newly Diagnosed Type 2 Diabetes Mellitus.
4	Endocrine	Diabetes mellitus	The comparison of CSF and serum GDF15 level	n=390	no T2DM	0.512	T2DM	1.154					GDF15 is significantly increased in overweight and type 2 diabetic patients and correlates positively with serum glucose and HbA1c	[55]	Characterization of growth differentiation factor 15 (GDF15) as a neurotropic adipokine permeable to the brain
4	Endocrine	Diabetes mellitus	To test whether circulating GDF-15 relates to metabolic traits and predicts peripheral neuropathy	n=283	Control	0.185	T2DM	0.606					Higher GDF-15 is associated with worse nerve-conduction parameters and identifies patients with peripheral neuropathy	[56]	Circulating Growth Differentiation Factor 15 Is Associated with Diabetic Neuropathy.
4	Endocrine	Diabetes mellitus	To determine whether circulating GDF-15 is associated with Diabetic peripheral neuropathy (DPN) presence and nerve-conduction indices (latency, amplitude, NCV), and whether GDF-15 serves as an independent predictor of DPN	n=162	no-DPN	0.366	T2DM	0.612					Higher GDF-15 is linked to worse nerve conduction (longer motor/sensory latencies; lower NCV) and is an independent predictor of DPN	[57]	Correlation Between Growth Differentiation Factor-15 and Peripheral Neuropathy in Patients with Type 2 Diabetes Mellitus
4	Endocrine	Diabetes mellitus	To assess whether circulating GDF-15 and GDF-15 gene polymorphisms are associated with T2DM risk and with glycemic indices	n=358	Control	0.878	T2DM	1.074					Positive correlations with FPG and HbA1c ; AUC 0.626 for discriminating T2DM vs controls.	[58]	Correlations between growth differentiation factor 15 (GDF-15) serum levels and gene polymorphism with type 2 diabetes mellitus
4	Endocrine	Diabetes mellitus	To test whether serum GDF-15 associates with Lower-extremity atherosclerotic disease (LEAD) independent of BMI.	n=376	no-LEAD	0.718	LEAD	0.91					Higher GDF-15 independently associated with LEAD	[59]	The association between serum growth differentiation factor-15 levels and lower extremity atherosclerotic disease is independent of BMI in T2DM
4	Endocrine	Diabetes mellitus	Plasma GDF15 level was analyzed to see the relation to peripheral artery disease in DM	n=174			DM	2.512					Elevated GDF15 was associated with worsening metabolic parameters and an increased risk of peripheral artery disease	[60]	The value of growth differentiation factor 15 as a biomarker for peripheral artery disease in diabetes patients
4	Endocrine	Hyperthyroidism	To test whether thyroid hormone excess elevates circulating GDF-15 and whether levels drop after treatment	n=239	Control	0.169	Hyperthyroidism	0.325					GDF-15 is significantly elevated in hyperthyroidism and falls toward normal after antithyroid therapy, independent of confounders.	[61]	Elevated Serum Growth Differentiation Factor 15 Levels in Hyperthyroid Patients
5	Heart	Acute coronary syndrome	To evaluate whether GDF-15 (and Tenascin-C) aid diagnosis/risk assessment of ACS among T2DM patients.	n=84	non-ACS	0.842	Acute coronary syndrome	1.15					GDF-15 is significantly higher in T2DM patients with ACS and correlates with CK-MB/CK-total, supporting diagnostic utility	[62]	Assessment of serum Tenascin-C and Growth Differentiation Factor-15 among type 2 diabetes mellitus patients with and without acute coronary syndrome.
5	Heart	Acute myocardial infarction	To evaluate whether admission GDF-15 improves early risk stratification and predicts 30-day mortality in cardiogenic shock	n=190			Acute myocardial infarction	7.662					Higher admission GDF-15 independently predicted 30-day mortality in CS; patients above the median had significantly higher mortality.	[64]	Growth-differentiation factor 15 and osteoprotegerin in acute myocardial infarction complicated by cardiogenic shock: a biomarker substudy of the IABP-SHOCK II-trial
5	Heart	Acute myocardial infarction	To compare GDF15 between AMI vs non-AMI and test correlation with coronary complexity	n=409	non-AMI	0.799	Acute myocardial infarction	1.122					GDF15 was higher in AMI and correlated with the SYNTAX score, remaining an independent	[63]	Association of GDF-15 and SYNTAX score in patients with acute myocardial infarction.
5	Heart	Acute myocardial infarction	To test whether circulating GDF-15 can predict the development of HF after AMI	n=170	non-HF after AMI	1.090	Heart Failure after AMI	1.35					GDF-15 is significantly higher in patients who developed HF after AMI	[65]	Plasma biomarkers for predicting heart failure in patients with acute myocardial infarction
5	Heart	Acute myocardial infarction	To test whether early (≤24 h) serum GDF15 predicts 12-month LV remodeling after STEMI (ST-segment elevation myocardial infarction).	n=97	No-LVR	1.998	Left ventricular remodeling	3.439					Admission GDF15 independently predicted LV remodeling	[66]	Relation of Growth-Differentiation Factor 15 to Left Ventricular Remodeling in ST-Segment Elevation Myocardial Infarction
5	Heart	Atrial fibrillation	To test whether baseline GDF15 predicts AF recurrence after ablation	n=150	AF-non-recurrence	1.062	AF-Recurrence	1.287					Higher pre-ablation GDF15 independently predicted AF recurrence and reflected atrial remodeling	[69]	The Predictive Value of Growth Differentiation Factor-15 in Recurrence of Atrial Fibrillation after Catheter Ablation.
5	Heart	Cardiovascular surgery	To test whether serum GDF-15 is associated with muscle mass/strength and sarcopenia	n=120	non-sarcopenia	0.948	Sarcopenia	1.63					Higher GDF-15 associates with lower skeletal muscle mass/strength and with sarcopenia	[70]	Association between serum GDF-15, myostatin, and sarcopenia in cardiovascular surgery patients
5	Heart	Cardiovascular surgery	To examine whether pre-op GDF15 stratifies risk in obese vs. non-obese cardiac-surgery patients.	n=80	non-obase	1.207	Obase	1.736					Pre-op GDF15 was higher in obese patients and associated with worse peri-operative profiles, supporting risk stratification	[71]	Serum GDF15, a Promising Biomarker in Obese Patients Undergoing Heart Surgery
5	Heart	Cardiovascular surgery	To evaluate whether preoperative GDF-15 helps predict 30-day mortality/morbidity after cardiac surgery	n=145			Patients	1.851					Higher preoperative GDF-15 was associated with increased short-term adverse outcomes after surgery	[72]	Preoperative Serum GDF-15, Endothelin-1 Levels, and Intraoperative Factors as Short-Term Operative Risks for Patients Undergoing Cardiovascular Surgery.
5	Heart	Cardiovascular surgery	To determine whether preoperative serum GDF-15 reflects renal dysfunction and muscle wasting/sarcopenia in cardiovascular surgery candidates.	n-72			Patients	1.676					Higher preoperative GDF-15 tracked lower eGFR and reduced thigh-muscle thickness/strength; it independently marked renal dysfunction and muscle wasting	[73]	Growth Differentiation Factor-15 (GDF-15) Is a Biomarker of Muscle Wasting and Renal Dysfunction in Preoperative Cardiovascular Surgery Patients.
5	Heart	Cardiovascular surgery	GDF15 was analyzed to predict heart failure in cardiac surgery	n=127			Patients	1.28					Higher serum GDF15 level related to high heart failure after cardiac surgery	[74]	Prediction of Japan Score and Society of Thoracic Surgeons Risk Scores for Patients Undergoing Cardiovascular Surgery by Serum Growth Differentiation Factor-15 and Endothelin-1 Levels
5	Heart	Coronary artery disease	To assess whether serum GDF-15 is elevated in CAD, relates to angiographic severity (Gensini score), and aids CAD discrimination (ROC).	n=201	Control	0.506	Coronary artery disease	2.907					GDF-15 was markedly higher in CAD, correlated strongly with disease severity (Spearman r = 0.85), and showed excellent discrimination (AUC = 0.96) with an optimal cutoff ≈ 0.951 ng/mL (~92% sensitivity, 80% specificity)	[67]	Increased Serum Level of Growth Differentiation Factor 15 (GDF-15) is Associated with Coronary Artery Disease.
5	Heart	Coronary artery disease	Serum GDF15 level was analyzed to see the prognosis of patients who underwent CABG.	n=82	Without adverse events	1.997	Adverse cardiovascular events	2.59					Cardiac death, hospitalization with HF, acute ischemic events requiring unplanned revascularization, or stroke was highre in patients with GDF15 level higher than 2.064	[75]	Predictors of Adverse Cardiovascular Events After CABG in Patients with Previous Heart Failure
5	Heart	Heart failure	To determine which factors—including serum GDF-15—are associated with 1-year all-cause mortality in this high-risk cohort	n=315	Survival	0.505	Non-Survival	1.618					Higher GDF-15 was independently associated with greater 1-year mortality	[76]	Growth differentiation factor-15 and routine laboratory parameters are associated with one-year mortality in patients with end-stage heart failure undergoing heart transplantation evaluation
5	Heart	Heart failure	To compare diagnostic utility of GDF-15 (with other markers) in HFpEF vs other HF entities vs controls.	n=252	Control	0.561	HFpEF	0.838	ICM	0.721	DCM	0.639	GDF-15 was significantly higher in all HF groups vs controls and showed good diagnostic performance for HFpEF	[77]	Expression of the Novel Cardiac Biomarkers sST2, GDF-15, suPAR, and H-FABP in HFpEF Patients Compared to ICM, DCM, and Controls
5	Heart	Heart failure	To assess prognostic value of GDF-15 for cardiovascular events in HFpEF	n=149	LVDD(no HF)	3.091	HFpEF	4.215					Higher baseline GDF-15 predicted more cardiovascular events and independently improved risk stratification in HFpEF.	[78]	Growth differentiation factor-15 is a useful prognostic marker in patients with heart failure with preserved ejection fraction (HFpEF).
5	Heart	Heart failure in pediatric patients	To test whether serum GDF-15 reflects heart-failure severity and the oxygen demand–supply balance in unrepaired shunt CHD.	n=69	unrepaired CHD	0.242	Infra-tricuspid shunt subgroup	0.401	Supra-tricuspid shunt subgrou	0.171			GDF-15 rose with worse clinical HF (modified Ross; AUC 0.992 at 0.564 ng/mL cut-off) and correlated with mean PAP, oxygen extraction, and NT-proBNP—supporting GDF-15 as a diagnostic/severity biomarker in unrepaired pediatric shunt CHD	[78]	Growth differentiation factor 15 as a useful biomarker of heart failure in young patients with unrepaired congenital heart disease of left to right shunt
5	Heart	Hypertension	To test whether serum GDF-15 is associated with a non-dipping BP pattern in newly diagnosed essential hypertension without LVH and whether it can aid discrimination	n=157	Control	0.494	Dipper HT	0.514	Non-dipper HT	0.558			GDF-15 was significantly higher in non-dippers; in multivariable analysis, GDF-15 and nighttime diastolic BP independently associated with non-dipping, and a cutoff of ~0.525 ng/mL yielded 56.7% sensitivity / 72.4% specificity (AUC 0.676).	[81]	Association between Growth Differentiation Factor 15 and Non-Dipping Circadian Pattern in Patients with Newly Diagnosed Essential Hypertension.
5	Heart	Pulmonary hypertension	To assess whether GDF-15 is altered in PH and helps characterize/stratify patients among PH subgroups relative to other biomarkers.	n=88	Control	0.514	Pulmonary hypertension	1.829					GDF-15 was substantially elevated in PH compared with controls and contributed to biomarker	[80]	Analysis of Novel Cardiovascular Biomarkers in Patients With Pulmonary Hypertension
5	Heart	Unstable angina pectoris	To evaluate the diagnostic value of serum GDF-15 for USAP (Unstable angina pectoris) and its relationship with GRACE/TIMI risk scores.	n=111	Control	0.85	USAP	2.3					Serum GDF-15 was significantly higher in USAP than in stable angina or controls, supporting diagnostic utility, but did not correlate with GRACE or TIMI scores	[68]	Serum Levels of Growth Differentiation Factor-15 as an Inflammatory Marker in Patients with Unstable Angina Pectoris.
6	Kidney	Hemodialysis	To examine links between serum GDF15, carotid IMT, and mortality in HD patients.	n=132	Control	0.176	Hemodialysis	0.328					GDF15 is elevated in HD, correlates with carotid IMT, and independently predicts all-cause mortality	[82]	Association of Growth Differentiation Factor 15 with Mortality in a Prospective Hemodialysis Cohort
6	Kidney	Renal function	To examine how GDF-15 relates to eGFR and hemoglobin (and sarcopenia indices) in healthy aging	n-66	Healthy Older female	1.01							GDF-15 correlated negatively with eGFR and positively/independently with lower hemoglobin	[41]	Association of serum growth differentiation factor-15 with eGFR and hemoglobin in healthy older females.
7	Liver	Chronic hepatitis B	To predict incident HCC during long-term NUC treatment with suppressed HBV DNA.	n=242			CH-B	0.86					Higher GDF15 independently associated with increased HCC incidence; no HCC over 10 years in patients with none of the identified risk factors.	[19]	High serum growth differentiation factor 15 is a risk factor for the occurrence of hepatocellular carcinoma in chronic hepatitis B patients treated with nucleos(t)ide analogs.
7	Liver	Chronic hepatitis C	To stratify/predict incident HCC after DAA-induced HCV elimination.	n=1606			CH-C	1.35					Higher baseline GDF15 (≈≥1.35 ng/mL) identified patients at greater post-SVR HCC risk in both cohorts.	[18]	Serum growth differentiation factor 15 predicts hepatocellular carcinoma occurrence after hepatitis C virus elimination.
7	Liver	Chronic hepatitis C	To evaluate whether serum GDF15 can non-invasively indicate presence and degree of liver fibrosis in chronic HCV	n=85	Control	0.11	CH-C	0.41					Serum GDF15 is significantly higher in HCV with fibrosis versus controls and correlates with fibrosis severity, supporting its use as a fibrosis biomarker.	[83]	Growth differentiation factor 15: an emerging diagnostic biomarker of liver fibrosis in chronic hepatitis C patients
7	Liver	Chronic hepatitis C	To evaluate serum GDF15 (with AFP and ferritin) as a non-invasive biomarker to diagnose advanced liver disease (cirrhosis, HCC) in chronic HCV.	n=80	Control	0.7	CH-C	1.41	LC	5.2	HCC	6.49	Serum GDF15 is markedly elevated in LC and HCC (and moderately in CHC) versus controls, supporting its use—especially alongside AFP—as a serum marker for cirrhosis/HCC in chronic HCV.	[84]	Significance of growth differentiation factor 15 in chronic HCV patients.
7	Liver	HCV-related cirrhosis	To predict liver-related adverse outcomes after viral elimination.	n=196			LC	2.6					Elevated baseline GDF15 post-SVR signaled worse prognosis (hepatic decompensation and related events).	[20]	Growth Differentiation Factor 15 Can Be a Novel Biomarker to Predict the Prognosis of Patients With Hepatitis C Virus Cirrhosis After Virus Elimination
7	Liver	MASLD	The comparison of GDF15 level between HIV patients with NAFLD and without NAFLD	n=177			HIV with MAFLD	0.859	HIV without MAFLD	0.744			GDF15 may aid in identifying people with HIV at risk of steatotic liver disease.	[86]	Circulating fibroblast growth factor 21 and growth differentiation factor 15 are associated with severity of hepatic steatosis in people with HIV
7	Liver	MASLD	To identify MASLD patients at high risk for liver cancer/events, independent of fibrosis stage.	n=1095			MASLD	1.23					GDF15 ≥1.75 ng/mL strongly enriched future liver cancer cases across cohorts, outperforming fibrosis stage alone.	[17]	Serum growth differentiation factor 15 is a novel biomarker with high predictive capability for liver cancer occurrence in patients with MASLD regardless of liver fibrosis.
7	Liver	MASLD	Protein was comprehensively analyzed to identify the protein elevated in MASH patients	n=484	MASLD without MASH	0.946	MASH	1.314					GDF15 was identified one of elevated proteins in MASH compared with MASLD witout MASH	[85]	Serum protein risk stratification score for diagnostic evaluation of metabolic dysfunction-associated steatohepatitis
8	Infection	COVID-19	To assess whether serum GDF-15 levels in children with COVID-19 are associated with disease severity, cardiac involvement	n=144	Control	0.114	COVID19-survivor	0.57	COVID19-non survivor	1.556			Higher GDF-15 tracks with greater clinical severity, ICU admission, cardiac involvement, and mortality	[89]	Serum growth differentiation factor-15 (GDF-15) is a biomarker of cardiac manifestations in childr
8	Infection	COVID-19	To test whether serum GDF-15 (with FGF-21) measured in the acute phase can predict later long-COVID symptoms in non-hospitalized patients	n=197			Acute COVID19 only	0,610	Long COVID in acute phase	1.12			Higher acute-phase GDF-15 is associated with having long-COVID symptoms at ~3 months	[87]	Growth Differentiation Factor-15 Is Considered a Predictive Biomarker of Long COVID in Non-hospitalized Patients
8	Infection	COVID-19	GDF-15 is a known severity/poor-outcome marker in COVID-19; they also included GDF-15 in multivariable models for survival prediction	n=152	Non-Fetal	4.354	Fetal	9.166					Higher GDF-15 associated with fatal outcome; in multivariable logistic regression GDF-15 remained an independent positive predictor	[88]	Prognostic Value of Catestatin in Severe COVID-19: An ICU-Based Study
8	Infection	Sepsis	Examining the prognostic significance of serum GDF-15 levels in ICU patients suffering from sepsis.	n=208	Survival	2.35	Non-Survival	3.27					Serum GDF15 can be potential application value as prognostic markers for sepsis.	[90]	Predictive Value of Combined Detection of Serum LGALS3BP and GDF-15 for the Prognosis of
8	Inflammation	IgG4-related disease	To test whether circulating GDF-15 reflects disease presence and fibrotic/organ involvement in IgG4-RD	n=116	Control	0.361	IgG4-Related disease	1.121					Serum GDF-15 was markedly elevated in IgG4-RD and independently associated with retroperitoneal fibrosis and parotid involvement	[91]	Association of serum levels of fibrosis-related biomarkers with disease activity in patients with IgG4-related disease
8	Inflammation	Kawasaki disease	To assess whether GDF-15 is elevated in KD and whether it predicts coronary artery lesions and IVIG non-response.	n=161	Control	0.662	Kawasaki-disease	1.825					GDF-15 was markedly higher in KD and independently predicted coronary artery lesions and IVIG non-response.	[92]	Increased concentrations of growth differentiation factor-15 in children with Kawasaki disease.
8	Inflammation	Rheumatoid arthritis	To evaluate whether serum GDF-15 reflects RA disease activity.	n=155			Rheumatoid arthritis-High activity	0.989	Rheumatoid arthritis-Low activi	0.349	Rheumatoid arthritis-Remission	0.349	GDF-15 strongly tracked disease activity	[93]	The association between serum levels of growth differentiation factor-15 and rheumatoid arthritis activity
8	Inflammation	Sjögren's syndrome	To test whether serum GDF15 distinguishes pSS from healthy controls and tracks extra-glandular involvement and disease activity	n=212	Control	0.672	Sjögren's syndrome	1.19					Serum GDF15 is elevated in pSS, increases with the number of extra-glandular manifestations, correlates with ESSDAI, and shows fair discrimination for pSS vs controls and active vs non-active disease.	[95]	Serum growth differentiation factor 15 associates with extra-glandular manifestations and disease activity of primary Sjögren's syndrome.
8	Inflammation	Systemic lupus erythematosus	Serum GDF15 level was analyze to examine risk for vein wall thickness in SLE	n=68	SLE without APS	0.2111	SLE with APS	1.323					Serum GDF15 level reflected by increased vein wall thickness (VWT) in SLE patients	[98]	A new characteristic of systemic lupus erythematosus: subclinical vein involvement—an in-depth biochemical and imaging study
8	Inflammation	Systemic lupus erythematosus	To test whether circulating GDF-15 distinguishes SLE from healthy controls and reflects disease activity/renal involvement	n=814	Control	0.153	SLE with high activity	0.727	SLE with low activity	0.306			Circulating GDF-15 is markedly elevated in SLE, tracks with disease activity and hematuria, and shows strong diagnostic discrimination vs healthy controls	[96]	GDF-15: A Potential Biomarker and Therapeutic Target in Systemic Lupus Erythematosus
8	Inflammation	Systemic lupus erythematosus	To evaluate the diagnostic and prognostic value of circulating GDF-15 in SLE-PAH (Systemic lupus erythematosus–associated pulmonary arterial hypertension)	n=148	Control	0.442	SLE-PAH	1.112	SLE without PAH	0.81			Higher in SLE-PAH vs both comparison groups	[97]	The diagnostic and prognostic value of growth differentiation factor-15 in systemic lupus erythematosus-associated pulmonary arterial hypertension
8	Inflammation	Systemic sclerosis	To evaluate whether serum GDF-15 reflects presence and severity of lung involvement in SSc.	n=244	Control	0.449	Systemic sclerosis	1.603					Serum GDF-15 is significantly elevated in SSc—especially with Interstitial lung disease (ILD) and honeycombing—supporting its use as a circulating marker of pulmonary involvement/severity	[99]	Growth Differentiation Factor 15, a Marker of Lung Involvement in Systemic Sclerosis, Is Involved in Fibrosis Development but Is Not Indispensable for Fibrosis Development.
8	Inflammation	Systemic sclerosis	To compare serum GDF15 between SSc and controls and relate it to clinical severity/ILD	n=95	Control	1.25	Systemic sclerosis	1.7					Serum GDF15 is higher in SSc—especially diffuse cutaneous and ILD cases—and correlates with skin score, supporting its role as a severity biomarker.	[100]	Growth Differentiation Factor-15 (GDF-15) Level and Relation to Clinical Manifestations in Egyptian Systemic Sclerosis Patients: Preliminary Data.
8	Inflammation	Spondyloarthritis (SpA)	To determine whether circulating GDF-15 associatesx with inflammation and structural damage (bone erosion) in SpA.	n=150	Control	0.191	Spondyloarthritis	0.504					GDF-15 was higher in SpA, correlated with CRP, and values ≥~502 pg/mL predicted MRI-defined bone erosion, supporting GDF-15 as a damage/inflammation-linked biomarker.	[101]	Increased serum levels of MIC1/GDF15 correlated with bone erosion in spondyloarthritis: A pilot study
9	Other_Lung	COPD	To evaluate whether serum GDF-15 can predict malnutrition in AECOPD (acute exacerbation of COPD)	n=142	Non-malnutrition	0.952	Malnutrition	1.117					Higher in malnutrition and independently associated with malnutrition (O	[151]	Serum growth differentiation factor 15 as a biomarker for malnutrition in patients with acute exacerbation of chronic obstructive pulmonary disease
9	Other_Lung	COPD	To determine whether circulating GDF-15 can identify/predict sarcopenia in COPD	n=235	COPD without sarcopenia	0.269	COPD with sarcopenia	0.438					Higher GDF-15 is linked to worse muscle status	[152]	Growth Differentiation Factor-15 as a Biomarker for Sarcopenia in Patients With Chronic Obstructive Pulmonary Disease
9	Other_Lung	Neonatal respiratory distress syndrome	To assess the serum GDF15 level in infant with RDS and w/o RDS	n=90	without RDS	0.71pg/mL	RDS	3.19pg/mL					Serum GDF15 was higher in infant with RDS than those without RDS	[153]	Association of macrophage migration-inhibitory factor gene and growth differentiation factor 15 gene polymorphisms and their circulating levels with respiratory distress syndrome among preterm neonates
9	Other_Blood	Severe aplastic anemia	To assess whether circulating GDF-15 relates to iron overload and erythropoietic activity (hepcidin, EPO, iron indices) in SAA.	n=53	Control	0.658	SAA (transfusion-dependent)	0.942	SAA (transfusion-independent)	0.744			GDF-15 was highest in transfusion-dependent SAA and associated with lower hepcidin and greater iron loading, supporting GDF-15 as a marker of ineffective erythropoiesis/iron overload in SAA.	[154]	Transforming growth factor 15 increased in severe aplastic anemia patients.
9	Other_Blood	Transfusion-dependent β-thalassemia (TDβT)	To assess whether serum GDF-15 is elevated in TDβT and whether it is associated with subclinical atherosclerosis, indexed by carotid intima–media thickness	n=91	Control	0.256	TDβT	1.84					GDF-15 was a significant predictor associated with atherosclerosis in TDβT	[155]	GDF‑15 is associated with atherosclerosis in adults with transfusion‑dependent beta‑thalassemia
9	Other_Brain	Acute cerebral infarction	To test whether GDF15 (with CXCL-16/PLA-2) reflects stroke severity and treatment response.	n=137	Control	0.87	Stroke	1.41					GDF15 is higher in stroke, scales with NIHSS/infarct size, and decreases after treatment—supporting monitoring utility.	[156]	Changes and significance of serum CXCL-16, GDF-15, PLA-2 levels in patients with cerebral infarction.
9	Other_Brain	Parkinson's disease	To test diagnostic value and association with motor severity in PD.	n=192	Control	0.288	Parkinson`s disease	0.573					Serum GDF15 was elevated in PD, correlated with UPDRS-III and disease duration, and showed good diagnostic discrimination	[157]	Serum Growth Differentiation Factor 15 in Parkinson Disease
10	Malignant	Acute myeloid leukemia	To evaluate whether serum GDF-15 levels differ between AML and controls and whether they carry prognostic information.	n=86	Control	0.065	AML	0.55					Serum GDF-15 is markedly higher in AML than controls and associates with adverse features, supporting its potential as a prognostic biomarker	[148]	Prognostic Impact of Serum Growth Differentiation Factor 15 Level in Acute Myeloid Leukemia Patients.
10	Malignant	Breast cancer	To evaluate whether serum GDF-15 can diagnose (especially early-stage) breast cancer—alone and combined with CA15-3	n=167	Control	0.385	Breast cancer	0.625					Serum GDF-15 was significantly higher in breast cancer than controls	[102]	A Combined Analysis of Serum Growth Differentiation Factor-15 and Cancer Antigen 15-3 Enhances the Diagnostic Efficiency in Breast Cancer.
10	Malignant	Breast cancer	To test whether baseline serum GDF-15 is associated with risk of treatment-related cardiotoxicity	n=103	No cardiotoxicity	0.71	Cardiotoxicity	1.048					Higher baseline GDF-15 associates with greater cardiotoxicity risk	[103]	GDF-15 is a potential candidate biomarker for an elevated risk of cardiotoxicity in breast cancer patients receiving neoadjuvant dual anti-HER2 therapy
10	Malignant	Colorectal cancer	To assess diagnostic/screening value vs CEA and relation to metastasis/recurrence	n=987	Control	0.36	CRC	0.859					GDF15 outperformed CEA for CRC detection and rose with liver metastasis/recurrence, supporting combined use with CEA	[104]	Circulating MIC-1/GDF15 is a complementary screening biomarker with CEA and correlates with liver metastasis and poor survival in colorectal cancer.
10	Malignant	Colorectal cancer	To evaluate GDF-15 (and TFF3) as serum markers for detecting CRC and predicting lymph-node metastasis	n=300	Control	0.639	CRC	2.03					Serum GDF-15 is markedly higher in CRC vs controls (AUC = 0.897) and higher in LNM than non-LNM CRC, supporting its utility for CRC detection and metastasis risk.	[105]	Identification of Serum Biomarkers for Colorectal Cancer Metastasis Using a Differential Secretome Approach.
10	Malignant	Colorectal cancer	To test whether serum GDF15 is higher when adenomas/CRC are present and whether it falls after adenoma excision.	n=272	Control	0.609	CRC	1.107	Adenomatous polyps	0.723	Hyperplastic polyps	0.582	Serum GDF15 rose stepwise from controls to adenomas to CRC	[106]	Macrophage inhibitory cytokine-1/growth differentiation factor-15 as a predictor of colonic neoplasia.
10	Malignant	Hepatocellular Carcinoma	The prognostic factor after HAIC	n=150			HCC	7.16					High GDF15 level patients had shorter OS and PFS after HAIC in HCC	[108]	Serum GDF15 level as predictive biomarker of clinical outcome in patients with unresectable hepatocellular carcinoma treated with hepatic arterial infusion chemotherapy
10	Malignant	Hepatocellular Carcinoma	To evaluate whether serum GDF15 helps diagnose LC/HCC and improves performance versus/with AFP.	n=1014	Control	0.31	CHC	2.13	LC	6.51	HCC	6.06	Association of Serum Level of Growth Differentiation Factor 15 with Liver Cirrhosis and Hepatocellular Carcinoma.	[107]	Association of Serum Level of Growth Differentiation Factor 15 with Liver Cirrhosis and Hepatocellular Carcinoma
10	Malignant	Lung cancer	To assess diagnostic utility and to monitor chemotherapy response	n=160	Control	0.564	Lung cancer	1.395					Serum GDF-15 was higher in lung cancer and changed with treatment	[109]	Value of Growth/Differentiation Factor 15 in Diagnosis and the Evaluation of Chemotherapeutic Response in Lung Cancer
10	Malignant	Melanoma	To test whether serum GDF-15 identifies UM patients with (early) clinically detectable metastases and outperforms routine tests.	n=206	Control	1.09	Metastatis Melanoma	10.53					Serum GDF-15 was markedly higher in metastatic UM versus non-metastatic UM and controls (est. sensitivity 83%, specificity 78%) and often rose months before imaging detected metastases	[110]	GDF-15: a novel serum marker for metastases in uveal melanoma patients.
10	Malignant	Melanoma	To assess whether serum GDF-15 improves overall-survival risk stratification and surveillance utility in advanced melanoma.	n=761			Melanoma (StageIII)	0.9	Melanoma (Stagge IV)	1.5			Higher serum GDF-15—especially ≥1.5 ng/mL—was independently associated with worse overall survival (notably in tumor-free stage III and unresectable stage IV), supporting its role as a prognostic/surveillance biomarker in melanoma.	[112]	High GDF-15 Serum Levels Independently Correlate with Poorer Overall Survival of Patients with Tumor-Free Stage III and Unresectable Stage IV Melanoma
10	Malignant	Melanoma	To evaluate serum GDF-15 (with BAFF and OPN) for detecting melanoma metastases and assess its kinetics before imaging diagnosis.	n=196	Control	0.64	Metastatis Melanoma	13.75					GDF-15 (cut-off 1209 pg/mL = 1.209 ng/mL) strongly discriminated metastatic cases and, combined with BAFF+OPN, improved detection (AUC up to 0.802) with rising levels up to two years pre-diagnosis	[111]	Evaluation of a Three-Marker Panel for the Detection of Uveal Melanoma Metastases: A Single-Center Retrospective Analysis.
10	Malignant	NSCLC, RCC, melanoma	To test prognostic significance of serum GDF-15 levels in patients undergoing immunotherapy	n=85			Solid cancer	26.4					High levels of GDF-15 were associated with worse survival outcomes and a higher likelihood of cancer-related cachexia	[145]	Impact of Serum GDF-15 and IL-6 on Immunotherapy Response in Cancer: A Prospective Study
10	Malignant	Oral squamous cell carcinoma	To test whether serum GDF15 distinguishes leukoplakia/OSCC from healthy controls and reflects tumor burden/prognosis.	n=114	Control	0.385	OSCC	1.351					Serum GDF15 is significantly elevated in leukoplakia and OSCC, falls after tumor resection, and was useful for discrimination at a 346.9 ng/L	[149]	Elevated level of serum growth differentiation factor 15 is associated with oral leukoplakia and oral squamous cell carcinoma.
10	Malignant	Oral squamous cell carcinoma	To test GDF-15 as a blood biomarker for OSCC diagnosis and prognosis and to assess perioperative change.	n=95	Control	0.501	OSCC	1.451					GDF-15 was significantly higher in OSCC than controls (AUC 0.943) and fell after tumor resection.	[150]	GDF 15 as an anti-apoptotic, diagnostic and prognostic marker in oral squamous cell carcinoma
10	Malignant	Pancreatic cancer	To define a serum GDF15 cut-off and examine links to cachexia symptoms and tumor signaling activity.	n=120	Benign pancreas disease	1.177	Pancreatic cancer	2.9					Higher GDF15 marks APC patients with worse performance, anorexia, inflammatory/tumor burden and activated pJNK/pAkt signaling, implicating GDF15 in cachexia biology.	[113]	Clinical and Tumor Characteristics of Patients with High Serum Levels of Growth Differentiation Factor 15 in Advanced Pancreatic Cancer.
10	Malignant	Pancreatic cancer	To assess MIC-1/GDF15 (with ADH and CA19-9) for diagnosing pancreatic cancer and improving early detection.	n=70	Control	1.93	Pancreatic cancer	4.46					Serum MIC-1/GDF15 was significantly elevated in pancreatic cancer and showed strong diagnostic performance, especially when combined with CA19-9.	[114]	Evaluation of circulating ADH and MIC-1 as diagnostic markers in Egyptian patients with pancreatic cancer.
10	Malignant	Pancreatic cancer	To assess whether serum GDF15 (alone/with CA125) differentiates PDAC from chronic pancreatitis.	n=63			Pancreatic cancer	7.695	Chronic pancreatitis	2.248			Serum GDF15 is markedly higher in PDAC than in chronic pancreatitis, and pairing GDF15 with CA125 improves diagnostic performance	[115]	Growth differentiation factor (GDF-15) concentration combined with CA125 levels in serum is superior to commonly used cancer biomarkers in differentiation of pancreatic mass.
10	Malignant	Pancreatic cancer	To assess diagnostic utility of serum MIC-1/GDF15 for PDAC	n=1472	Control	0.417	Pancreatic cancer	1.731	Chronic pancreatitis	1.299	Benign pancreatic tumor	0.808	MIC-1/GDF15 is significantly elevated in PDAC and improves early detection—especially when combined with CA19-9.	[116]	Macrophage inhibitory cytokine 1 (MIC-1/GDF15) as a novel diagnostic serum biomarker in pancreatic ductal adenocarcinoma
10	Malignant	Pancreatic cancer	To evaluate whether serum MIC-1/GDF15 helps detect premalignant pancreatic lesions and predict neoplastic tumours during high-risk screening.	n=120	Control	0.57	Pancreatic cancer	1.04					Baseline GDF15 was significantly higher in participants with neoplastic tumours and predicted neoplasia (AUC≈0.81), supporting its potential as a serological flag for further imaging in high-risk individuals	[117]	Macrophage inhibitory cytokine-1/growth differentiation factor-15 in premalignant and neoplastic tumours in a high-risk pancreatic cancer cohort.
10	Malignant	Pancreatic cancer	To evaluate whether serum MIC-1/GDF15 can detect pancreatic/periampullary cancers and how it compares with CA19-9 diagnostically.	n=325	Control	0.546	Pancreatic cancer	2.43	Chronic pancreatitis	1.36	Benign pancreatic tumor	0.94	Serum MIC-1/GDF15 is significantly elevated in PDAC and other periampullary cancers vs benign disease/healthy controls, performs similarly to CA19-9 on its own, and improves diagnostic accuracy when combined with CA19-9.	[118]	Serum Macrophage Inhibitory Cytokine 1 as a Marker of Pancreatic and Other Periampullary Cancers.
10	Malignant	Pancreatic cancer	To compare serum MIC-1/GDF-15 with CA19-9 (and other candidates) for diagnosing pancreatic cancer versus chronic pancreatitis and healthy controls.	n=150	Control	0.662	Pancreatic cancer	4.745					MIC-1/GDF-15 outperformed CA19-9 for cancer vs healthy (AUC 0.99 vs 0.78; p=0.003) but was similar for cancer vs chronic pancreatitis (AUC 0.81 vs 0.74); combining markers offered limited additional benefit	[119]	Serum Markers in Patients with Resectable Pancreatic Adenocarcinoma: Macrophage Inhibitory Cytokine-1 versus CA19-9.
10	Malignant	Prostate cancer	To test whether serum MIC-1/GDF15 improves discrimination of PC vs BPH/healthy and relates to PSA and Gleason score.	n=240	Control	0.37	Prostate Cancer	1.536	Benign prostatic hyperplasia	0.621			Serum MIC-1/GDF15 was significantly higher in PC than in BPH/healthy, correlated with Gleason score (r≈0.7), and—alone or with PSA—showed excellent diagnostic performance	[120]	Relevance of MIC-1 in the Era of PSA as a Serum-Based Predictor of Prostate Cancer: A Critical Evaluation
10	Malignant	Prostate cancer	To test whether serum MIC-1/GDF15 predicts the presence of bone metastases and future bone relapse in prostate cancer.	n=159	No relapse	0.988	Bone relapse	1.477					Serum MIC-1/GDF15 independently predicted current bone metastasis and higher baseline levels were associated with future bone relapse; it outperformed PSA and bone-turnover markers in ROC analysis	[121]	Serum macrophage inhibitory cytokine-1 concentrations correlate with the presence of prostate cancer bone metastases.
10	Malignant	Solid cancers (gastrointestinal and lung	To examine links between circulating GDF-15, anorexia, and muscle mass in cancer.	n=59	Control	0.55	Solid cancers	0.934					GDF-15 was higher in cancer—especially anorexic and GI-cancer patients—supporting its role in cancer-associated anorexia.	[122]	Association between Growth Differentiation Factor-15 (GDF-15) Serum Levels, Anorexia and Low Muscle Mass among Cancer Patients.
